# Sleep-physiological correlates of brachycephaly in dogs

**DOI:** 10.1007/s00429-023-02706-y

**Published:** 2023-09-24

**Authors:** Ivaylo Borislavov Iotchev, Zsófia Bognár, Katinka Tóth, Vivien Reicher, Anna Kis, Enikő Kubinyi

**Affiliations:** 1 Department of Ethology, Eötvös Loránd University, Budapest, Hungary; 2 Doctoral School of Biology, Eötvös Loránd University, Budapest, Hungary; 3 Institute of Cognitive Neuroscience and Psychology, Research Centre for Natural Sciences, Budapest, Hungary; 4 ELTE-ELKH NAP Comparative Ethology Research Group, Budapest, Hungary; 5 Developmental and Translational Neuroscience Research Group, Institute of Cognitive Neuroscience and Psychology, Research Centre for Natural Sciences, Budapest, Hungary; 6 MTA-ELTE Lendület “Momentum” Companion Animal Research Group, Budapest, Hungary; 7 ELTE NAP Canine Brain Research Group, Budapest, Hungary

**Keywords:** Animal models, Neuroanatomy, Spectral power, Sleep spindles, REM, Non-REM

## Abstract

The shape of the cranium is one of the most notable physical changes induced in domestic dogs through selective breeding and is measured using the cephalic index (CI). High CI (a ratio of skull width to skull length > 60) is characterized by a short muzzle and flat face and is referred to as brachycephaly. Brachycephalic dogs display some potentially harmful changes in neuroanatomy, and there are implications for differences in behavior, as well. The path from anatomy to cognition, however, has not been charted in its entirety. Here, we report that sleep-physiological markers of white-matter loss (high delta power, low frontal spindle frequency, i.e., spindle waves/s), along with a spectral profile for REM (low beta, high delta) associated with low intelligence in humans, are each linked to higher CI values in the dog. Additionally, brachycephalic subjects spent more time sleeping, suggesting that the sleep apnea these breeds usually suffer from increases daytime sleepiness. Within sleep, more time was spent in the REM sleep stage than in non-REM, while REM duration was correlated positively with the number of REM episodes across dogs. It is currently not clear if the patterns of sleep and sleep-stage duration are mainly caused by sleep-impairing troubles in breathing and thermoregulation, present a juvenile-like sleeping profile, or are caused by neuro-psychological conditions secondary to the effects of brachycephaly, e.g., frequent REM episodes are known to appear in human patients with depression. While future studies should more directly address the interplay of anatomy, physiology, and behavior within a single experiment, this represents the first description of how the dynamics of the canine brain covary with CI, as measured in resting companion dogs using a non-invasive sleep EEG methodology. The observations suggest that the neuroanatomical changes accompanying brachycephaly alter neural systems in a way that can be captured in the sleep EEG, thus supporting the utility of the latter in the study of canine brain health and function.

## Introduction

Brachycephaly, characterized by a relatively short head and flat face, is one of the most salient morphological changes imposed upon dogs by selective breeding. The extent to which the skull was shortened and the face flattened in some modern breeds is unmatched among wild canines and an accelerating trend in breeding practices [see, e.g., (Teng et al. 2016)]. The degree to which a dog's head shape is brachycephalic is measured with the cephalic index (CI), which is the ratio of skull width to length, thus higher in more brachycephalic animals. Some authors specifically define brachycephaly as CI exceeding a value of 60 [(Stone et al. 2016) skull width/length*100].

CI is associated with a wide variety of changes, some more predictable than others, observed across behavior, perception, and health. Brachycephalic dogs are more vulnerable to respiratory and cardiovascular disorders [reviewed in Packer and O'Neill (2021)], but perhaps more surprisingly, there is also physiological (McGreevy et al. 2004) and behavioral (Gácsi et al. 2009; Bognár et al. 2021) evidence for better visual capacity than most canines concerning acuity and binocular processing. One cluster of differences, in particular, is pushing for a thorough neuroscientific assessment of breed differences defined by CI. First, evidence is growing that the shortening of the canine skull is accompanied by anatomical changes (Schmidt et al. 2015; Czeibert et al. 2020; Rusbridge and Knowler 2021). Loss of white matter and cortical surface, unusually large ventricles, hydrocephaly, as well as hypoxia in the brain, which affect brain health and function. Second, there is currently a growing catalog of behavioral changes observed as a function of CI (Gácsi et al. 2009; Horschler et al. 2019; Bognár et al. 2021). At least some of the findings suggest that cognitive performance might be worse in brachycephalic dogs (Horschler et al. 2019).

There is currently no complete sketch of the path from anatomy to behavior. A step that is specifically missing is measuring activity in the living dog's brain as a function of CI. This is crucial for two reasons. For properly assessing the welfare implications of breeding for high CI, we need to understand how the causal chain from changes in appearance to changes in behavior unfolds on every level. A broader, but more theoretical concern is the fruitful ground offered by selective breeding for studying evolutionary principles. This was famously demonstrated in the farm fox experiment (Trut 1999), which helped sketch a scenario for the emergence of domestication. In the case of breeds characterized by differences in brain anatomy, selective breeding can be specifically applied to the study of brain evolution.

Recent advances in the field of canine neuro-cognition (Bunford et al. 2017) have resulted in measurement techniques suitable for recording dogs' brain activity in a fully non-invasive and, thus, ecologically valid manner. The perhaps most accessible of those methods is canine polysomnography (EEG measurement during sleep), since the relative absence of motor activity during sleep accounts for a low incidence of artifacts even in untrained animals. Over the last few years, research in dogs (Kis et al. 2017c; Iotchev et al. 2017, 2020a, b) has corroborated the notion that brain activity during sleep correlates with awake cognitive performance (Genzel et al. 2014), behavior (Carreiro et al. 2023), as well as affective and mood states (Kis et al. 2017b; Kiss et al. 2020). This either reflects sleep-specific contributions to information processing, e.g., sleep-dependent memory consolidation (Genzel et al. 2014), or the general state of mechanisms that manifest in both sleep and waking EEG (Chen et al. 2016). The present study will likewise employ sleep EEG recordings to see if parameters previously shown to relate to dog behavior and cognition are associated with canine brachycephaly.

So far, most human brain pathologies are reported to leave marks in the sleep-recorded EEG. They affect the spectral properties of the signal (Castelnovo et al. 2020; Stern 2020), the latency and duration of sleep stages, e.g., REM (Palagini et al. 2013), and the expression of transients like sleep spindles (Lopez and Hoffmann 2010; Merikanto et al. 2019) and K-complexes (Rodríguez-Labrada et al. 2019). However, a few studies have investigated how sleep EEG changes in direct response to anatomical and structural brain changes. The vast majority of work in humans either directly compares sleep quality (i.e., duration, efficiency, and subjective reports) to lesions (Babu Henry Samuel et al. 2022) and loss of gray matter (Grau-Rivera et al. 2020) or white matter (Bai et al. 2022), thus circumventing EEG. In other works, sleep EEG parameters are compared to psychiatric diagnosis (Keshavan et al. 1998; Palagini et al. 2013), thereby, in most cases, leaving out a direct assessment of anatomy. Of the few more deeply examined anatomical conditions, white-matter loss is of particular interest, since it is one of the reported anatomical correlates of brachycephaly in dogs (Schmidt et al. 2015). Sanchez et al. (2019, 2020) offer a few observations on how the sleep EEG signal in traumatic brain injury (TBI) patients changes in response to white-matter loss. They found sleep spindles to be relatively resilient, with only the intrinsic frequency of frontal spindles being negatively correlated with white-matter loss. White matter loss was also associated with an increase in power and peak-to-peak amplitude for the delta frequency band [0.5–4 Hz (Sanchez et al. 2019)]. Both effects were observed within the TBI populations, while there was no difference found in the comparison between TBI and healthy controls.

Sleep macrostructure, i.e., the duration of the REM and non-REM phases of sleep, can be specifically helpful regarding the earlier mentioned goal to model brain evolution in dog breeds. Macrostructure varies strongly between species (Zepelin et al. 2005), and some preliminary findings suggest that it may also differ between dogs and the closely related wolf (Reicher et al. 2022). However, results relating to macrostructure may not be easy to interpret in the absence of behavioral measures, since early development (Zepelin et al. 2005) and mood disorders (Palagini et al. 2013) might also account for a prolonged duration (and early onset) of the REM sleep stage.

In the present study, we investigate a range of sleep parameters (macrostructure, spectral power, and sleep spindles) as a function of CI. Two different, but not mutually exclusive, global effects are expected to result from high CI. First and straightforward, the reduction of the cortical surface and white matter around ventricles in high CI dogs may be an anatomical indicator of neuropathology. It can be thus expected to correlate with EEG markers of worse cognitive performance, i.e., low REM beta power, high REM delta power (Kis et al. 2017c), low sleep spindle density, and/or amplitude. Second, breeding for high CI may be driven by aiming for dogs with cute appearances and thus further enforces some of the juvenile features that increased during initial domestication (Trut 1999). It has been shown in various works [reviewed in Pörtl and Jung (2019)] that this juvenilization also expresses in physiology and may thus reflect in sleep measures, as well. One possibility is to find prolonged REM phases in the brachycephalic dog, since REM is abundant in the early development of some species and may, in fact, be a carry-over from fetal life (Zepelin et al. 2005). Under the latter hypothesis, we would also expect spectral properties of the non-REM sleep stage to be affected, as they rapidly change during early development [decrease in delta and increase in higher frequencies like beta, sigma, and theta until the dogs reach 14 months of age (Reicher et al. 2021)].

## Methods

### Ethical statement

According to the Hungarian regulations of animal experimentation, our non-invasive polysomnography research does not qualify as an animal experiment (‘1998. évi XXVIII. Törvény’ 3.§/9.—the Animal Protection Act). The Hungarian Scientific Ethical Committee of Animal Experiments has also issued a specific permission (under the number PE/EA/853–2/2016) for our non-invasive protocol. All owners volunteered to participate in the study and were informed about the procedure before the start of the recordings.

### Subjects

Polysomnographic EEG and CI from 92 dogs (48 ♀, mean age ± SD: 8.2 ± 3.4 years, age range: 1–14 years) were available for analysis in this study. Of these dogs, 38 (41.3%) are mixed breeds, while the remaining purebred animals belong to 27 different breeds. For all dogs, the mean CI ± SD was 53.3 ± 5.8. Only 21 dogs were reproductively intact (22.8%) and 4 dogs (4.3%) were of unknown reproductive status.

The EEG data were taken from a constantly growing database, and therefore, there is an overlap in subjects with the other studies from our group (Iotchev et al. 2019, 2020a).

### CI definition and measurement

**Fig. 1:**
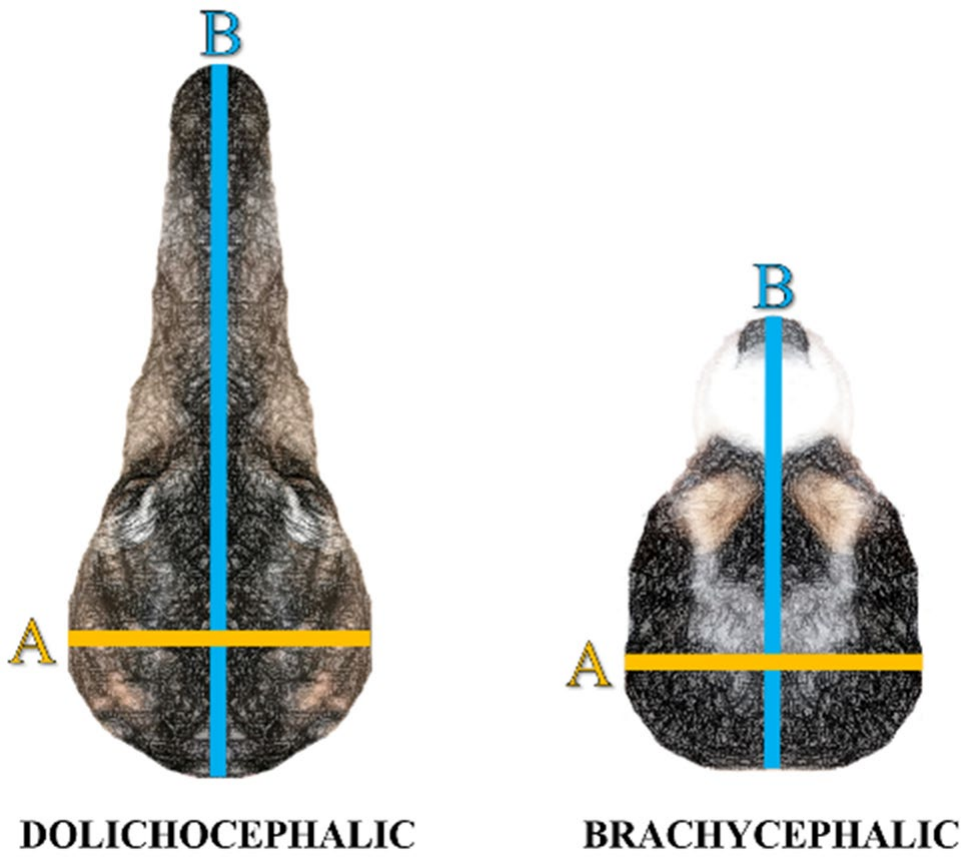
Calculation of the cephalic index (CI). CI is the ratio of the maximum width of the head (**A**) multiplied by 100, then divided by the head’s maximum length (**B**). CI is higher for brachycephalic dogs (common threshold value > 60)

**Fig. 2:**
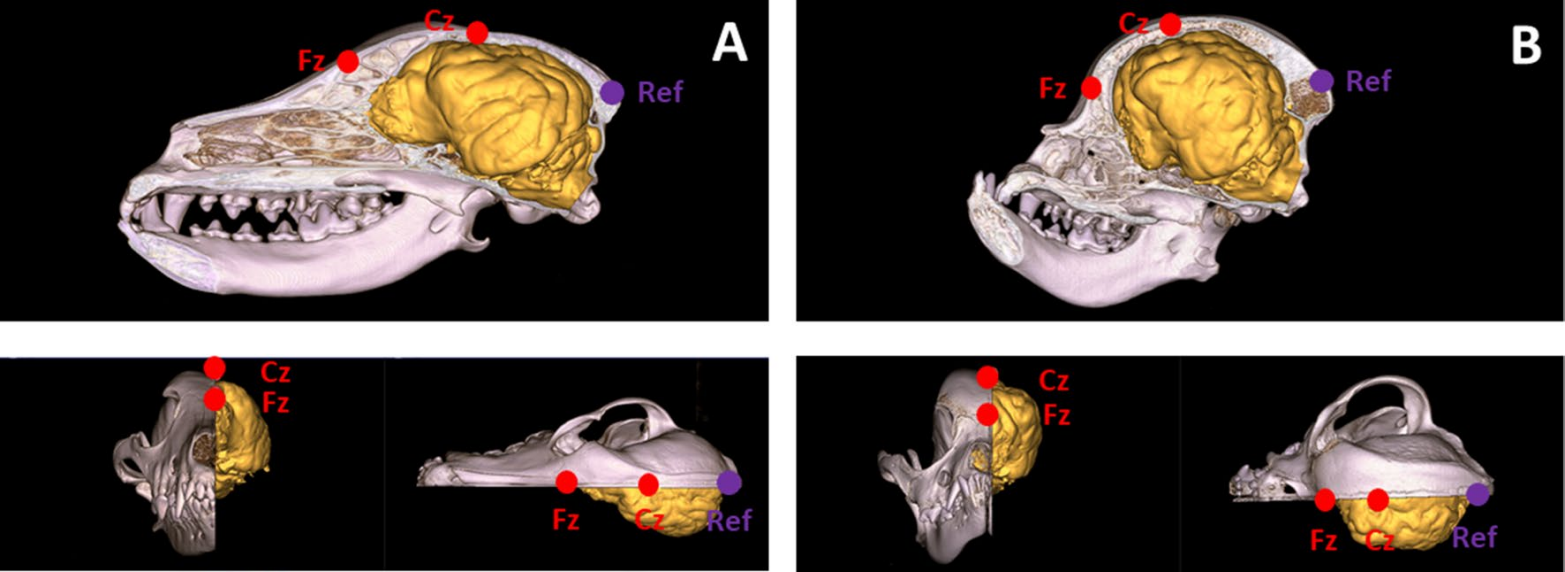
Electrode placement Fz and Cz (active electrodes, in red) and Ref (reference, in purple) in dolichocephalic dogs (**A**) and brachycephalic dogs (**B**). Possible implications for our measurements are elaborated on in the Discussion. Images are courtesy of Dr. Kálmán Czeibert

The CI was calculated as the ratio of the maximum width of the skull (from one zygomatic arch to the other) multiplied by 100 and divided by the skull's maximum length (from the nose to the occipital protuberance, see also Figs. 1 and 2). The CI of each dog was measured from photographs with the GIMP image editing program 2.2.13. (http://www.gimp.org/). The photographs were taken either when the dogs visited our laboratory for behavioral testing (Bognár et al. 2021) or at home by the owner (based on specific instructions). Each photograph was taken from the same angle (perpendicular to the top of the skull; see examples in Bognár et al. (2021). Although the distance of the camera to the top of the dogs' skull was not uniform, this did not affect the measurement, as the cephalic index is a ratio. The reliability of measuring the cephalic index from photographs was previously checked by comparison with a second, naïve coder (ICC: 0.91, *p* < 0.001) and using a caliper [ICC: 0.98, *p* < 0.001, originally reported in Bognár et al. (2021)].

### EEG implementation and analysis

The method for measuring polysomnography in dogs was first described by Kis et al. (2014); subsequent variations are discussed by Iotchev et al. (2019, 2020a). In all variations of the setup, there is an active frontal electrode, which is identically placed (Fz). In 82.6% of all dogs (76 animals), however, there was a second active electrode (Cz), placed centrally on the skull, between Fz and the occipital bone. The exact position of the Fz and Cz electrodes relative to the brain and skull is depicted in Fig. 2A for dolichocephalic dogs and Fig. 2B for brachycephalic dogs. Electrodes (both Fz and Cz if active) were referenced against the occipital bone. Due to the reference type, the setup is unipolar, but in 17.4% of the sample (16 dogs), only Fz was active. Other electrodes were placed on the left musculus temporalis (ground) and on the zygomiotica, for measuring eye movements (electrodes F7 and F8). Cardiac and respiratory frequencies, as well as muscle tone, were monitored to aid subsequent sleep-stage identification. In all set-ups, the type of electrode used was gold-coated Ag/AgCl fixed at the scalp surface with EC2 Grass Electrode Cream (Grass Technologies, USA). Impedance was kept below 15 kΩ. The electrode signals were collected and preprocessed with a 30-channel Flat Style SLEEP La Mont Headbox and an HBX32-SLP 32-channel preamplifier (La Mont Medical Inc., USA).

All recordings were first-time measurements, meaning that the animals were new to the sleeping laboratory, although the dogs were given a brief (5–10 min) free exploration of the room prior to electrode attachment. The recordings were exclusively afternoon measurements (starting time: from 12 to 6 pm). The intended recording duration based on the protocol was 3 h (mean ± SD: 168.6 ± 32.8 min). During a recording, the dog was alone in a darkened room with their owner. The owner was positioned on a mattress, and the dog could freely choose to settle down on the same mattress or on an adjacent rug; no restrictions were applied to the animals’ movement. Experimenters were only present in the sleeping area during electrode placement and detachment.

**Fig. 3:**
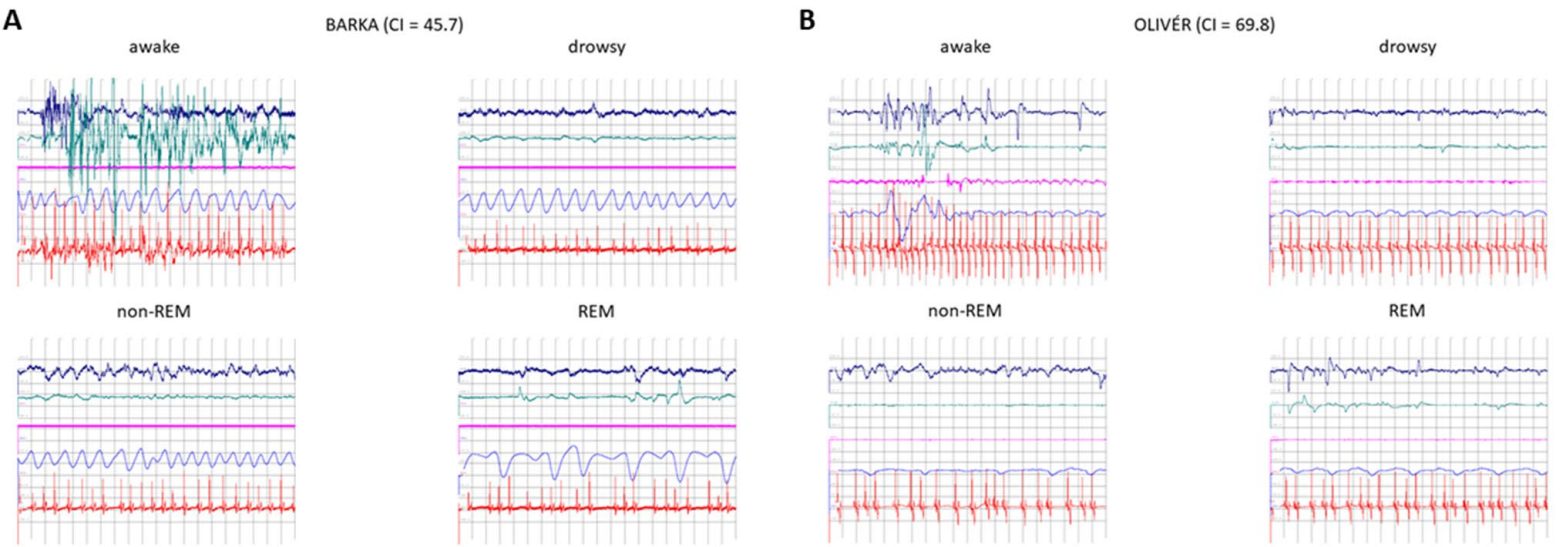
Polysomnographs of sleep stages in a dolichocephalic, low CI dog (**A**) and a brachycephalic, high CI dog (**B**). Channel order and color-coding: EEG trace (Fz exemplifies both active channels) - dark blue, eye-movements - dark turquoise, muscle tone - magenta, respiration - blue, heartbeat - red

Sleep-stage identification followed the criteria outlined by Kis et al. (2014) and was further validated by Gergely et al. (2020). In short, polysomnographic monitoring of the hindleg muscles, eye muscles, heartbeat, and the EEG were used to categorize the signal into wakefulness, drowsiness, REM, and non-REM. Categorization of the signal was performed separately for each epoch of 20 s length, while artifacts were identified within 4-s-long epochs. Wakefulness was defined by the presence of high-frequency and amplitude eye movements, elevated muscle tone, and a fast activity EEG signal. Drowsiness was scored when the amplitude and frequency of the eye movements decreased, and the muscle tone was attenuated compared to wakefulness, but EEG activity remained of predominantly high frequency. For non-REM classification, we required delta (1–4 Hz) to be at ≥ 15 μV, i.e., presenting a markedly slowed down activity compared to the other stages; eye movements to be absent or of very low amplitude, and muscle tone to be likewise low. In both drowsiness and non-REM, respiration was expected to be regular, while irregular respiration and heartbeat, complete muscle atonia, combined with fast, irregular EEG activity and rapid eye movements were required to categorize the signal as REM. In Fig. 3, we demonstrate an example for the polysomnography of each sleep stage in each of two dogs—one low CI, dolichocephalic animal (Barka, 2A) and a high CI, brachycephalic subject (Olivér, 2B).

A method for automatic sleep spindle detection was first introduced by Iotchev et al. (2017). It remained constant in subsequent studies, with the exception of the filter representation, which was changed in 2019 [(Iotchev et al. 2019) from discrete time zero-pole-gain to a second-order section] to account for the effects of different recording devices on the filter response of the EEG signal.

In the context of the present study, we now also introduce a new method for spectral density analysis. Since our previous work in the dog (Kis et al. 2017c; Reicher et al. 2022) made use of a locally distributed software for spectral data extraction (Fercio’s EEG Plus software, 2009–2022, developed by Ferenc Gombos), we argue that future replication efforts across research groups may benefit from implementing a more widely used software like Matlab. To this end, we devised a Matlab-based script for relative power extraction based on the spectrogram function therein. Indices for detecting artifact-free segments and identifying the sleep stage to which a segment corresponds were incorporated into the data prior to uploading it in Matlab. Following sleep-stage selection and artifact rejection, the signal was first filtered with a Butterworth (second-order section representation) filter (passband boundaries: 0.1–30 Hz, stopband boundaries: 0.05–35 Hz). The passband boundaries of the filter were chosen to match parameters common in both awake (Batterink and Paller 2017; Moser et al. 2021; Batterink and Zhang 2022) and sleep (Waterman et al. 1993; Lyamin et al. 2008; Batterink and Zhang 2022) EEG studies across human and non-human animals. Next, absolute power values were extracted with the spectrogram function in Matlab, specifying 4-s-long time-windows with 50% (2 s) overlap for a time–frequency analysis. These parameters match our earlier settings for calculating band-specific power in the dog (Kis et al. 2017c). As in our automated sleep spindle detection, zero-padding was applied to achieve a 0.1 Hz resolution. Thus, obtained power values were averaged across time-windows within each sleep-stage (REM versus non-REM), recording and dog and separately for the power bands alpha, beta, theta, and delta. As previously in the dog (Kis et al. 2017c) and wolf (Reicher et al. 2022), alpha was defined as 8–12 Hz, beta as 12–30 Hz, theta as 4–8 Hz, and delta as 1–4 Hz. Sigma [12–16 Hz (Kis et al. 2014) or 9–16 Hz (Iotchev et al. 2017)] was not analyzed, because we instead quantified sleep spindles as discrete events. After the power spectrum for each band had been averaged across time-windows, a second averaging across frequencies within the band of interest ensured a single final value for that band, sleep stage, and recording. Relative power in, e.g., REM alpha was the percent of absolute alpha power from the sum of REM alpha, REM beta, REM theta, and REM delta. Relative power values for the four bands of interest and from each sleep stage were subsequently used in our statistical analyses.

### Statistical analysis

Pearson correlations were used to compare CI with any of the sleep variables: duration of REM, non-REM, drowsiness, and wakefulness in minutes; relative power for alpha, beta, theta, and delta in each REM and non-REM; density, frequency, and amplitude of fast (≥ 13 Hz), slow (≤ 13 Hz), and generic (9–16 Hz) spindles. Correlations of CI with relative power in REM and non-REM were corrected for the duration of the respective sleep stage by adding the latter as a control variable in partial correlations. Possible confounds from age, sex, and reproductive status were tested in a series of control analyses inquiring if CI was correlated with age or different for male and female; intact and neutered dogs. The last two comparisons were conducted as independent samples t tests. All analyses were conducted in SPSS v25.

## Results

### Control analyses

Dogs of different ages were uniformly distributed among different head shapes, as evidenced by the lack of correlation between CI and age (*p* = 0.968). There was also no difference in average CI between sub-samples defined by sex (*p* = 0.241) or reproductive status (*p* = 0.602). The duration of the recordings was not correlated with CI (*p* = 0.438) which suggests that variations in this parameter cannot explain below results.

### Sleep-stage durations

**Fig. 4:**
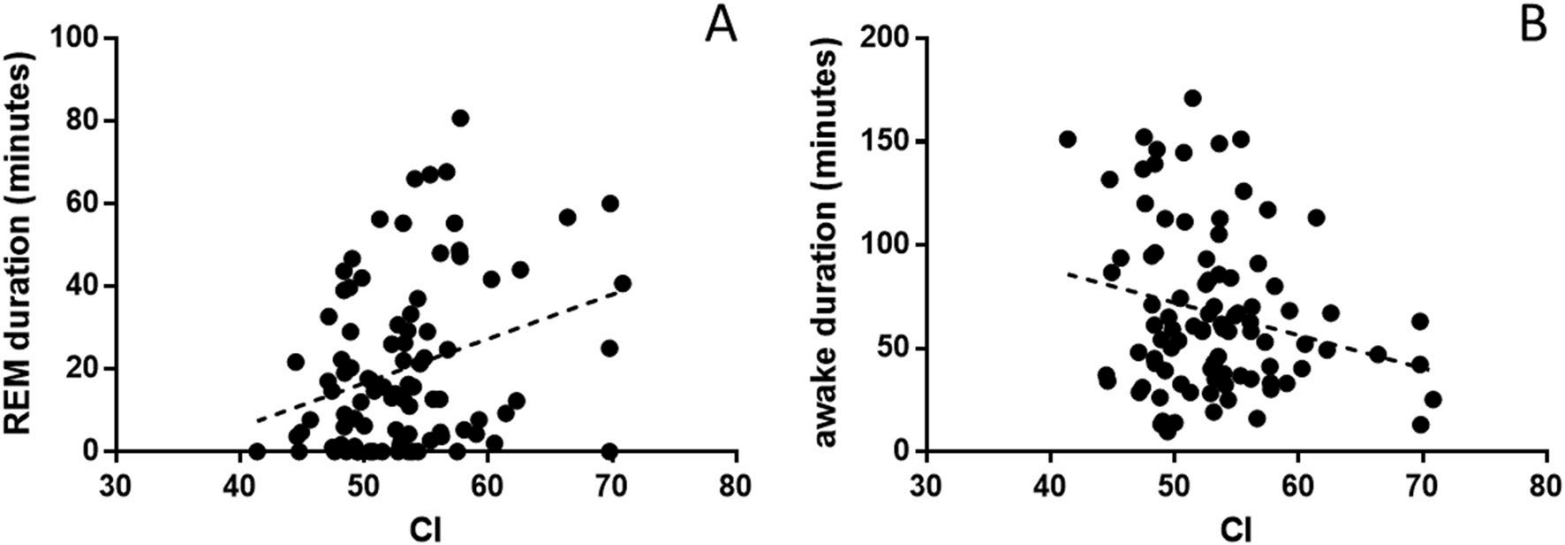
CI and sleep stage durations (in minutes). Correlations were significant with REM duration (**A**) and time spent awake (**B**)

CI was significantly positively correlated with time spent in REM (*r* = 0.307, *P* = 0.003, Fig. 4A) and negatively with time spent in wakefulness (*r* = − 0.233, *P* = 0.025, Fig. 4B).

Ten recordings were substantially shorter than intended by protocol (< 2 h). Removing these data points did not change the results: CI was again correlated with time spent in REM (*r* = 0.383, *P* < 0.001) and with time spent awake (*r* = − 0.275, *P* = 0.012).

CI was not linked to the number of awakenings (*P* = 0.570).

CI was positively correlated with a higher REM to non-REM ratio (*r* = 0.248, *P* = 0.020).

A positive link was observed between CI and relative time spent asleep when only REM and non-REM were included in the definition of sleep (*r* = 0.220, *P* = 0.035). The association was not significant when drowsiness was also counted as part of sleep (*P* = 0.099). Other correlations between CI and sleep macrostructure variables (non-REM and drowsiness duration) were not significant (*P* > 0.3).

### REM episodes

We also calculated the average duration of an REM episode by dividing the total time spent in REM by the number of transitions into REM. Average REM episode duration was not correlated with CI (*r* = 0.108, *P* = 0.378), nor was CI linked to the number of REM episodes (*r* = 0.152, *P* = 0.188). For the sample as a whole, however, total REM duration and the number of REM episodes were correlated (*r* = 0.770, *P* < 0.001).

### Relative power

**Fig. 5:**
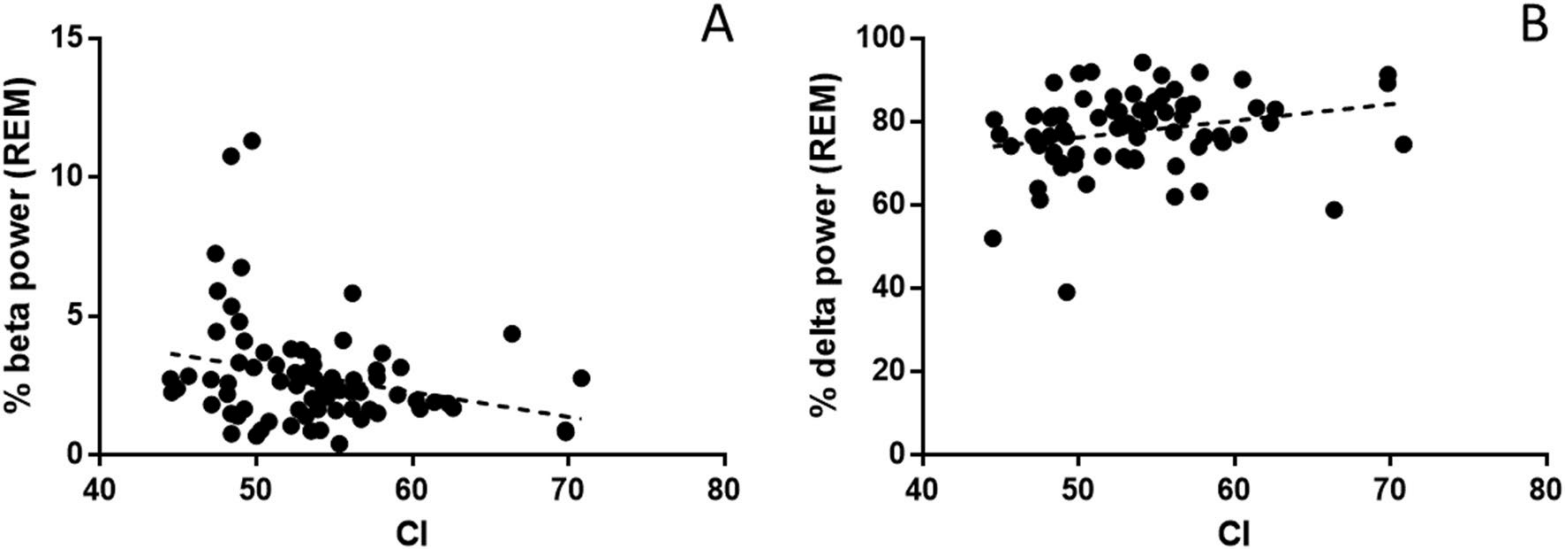
CI and relative power on Fz during REM. Correlations were significant for the beta (**A**) and delta (**B**) frequency bands

On Fz, during REM, relative beta (12–30 Hz) power was negatively correlated with CI (*r* = − 0.261, *P* = 0.025) and positively with delta (1–4 Hz) power (*r* = 0.238, *P* = 0.041). These results are summarized in Fig. 5. Correcting for REM duration with partial correlations, the effect remained significant for beta power (*r* = − 0.232, *P* = 0.049), but not delta power (*P* = 0.139). No other correlations were significant with CI on Fz (theta, 4–8 Hz and alpha, 8–12 Hz in REM; all bands in non-REM; *P* > 0.1).

On Cz, during REM, relative delta (1–4 Hz) power was positively correlated with CI (*r* = 0.257, *P* = 0.046). The effect was not significant after correcting for REM duration (*P* = 0.083). No other correlations were significant with CI on Cz (theta, 4–8 Hz; alpha, 8–12 Hz; beta, 12–30 Hz; in REM and all bands in non-REM; *P* > 0.05).

### Sleep spindles

**Fig. 6:**
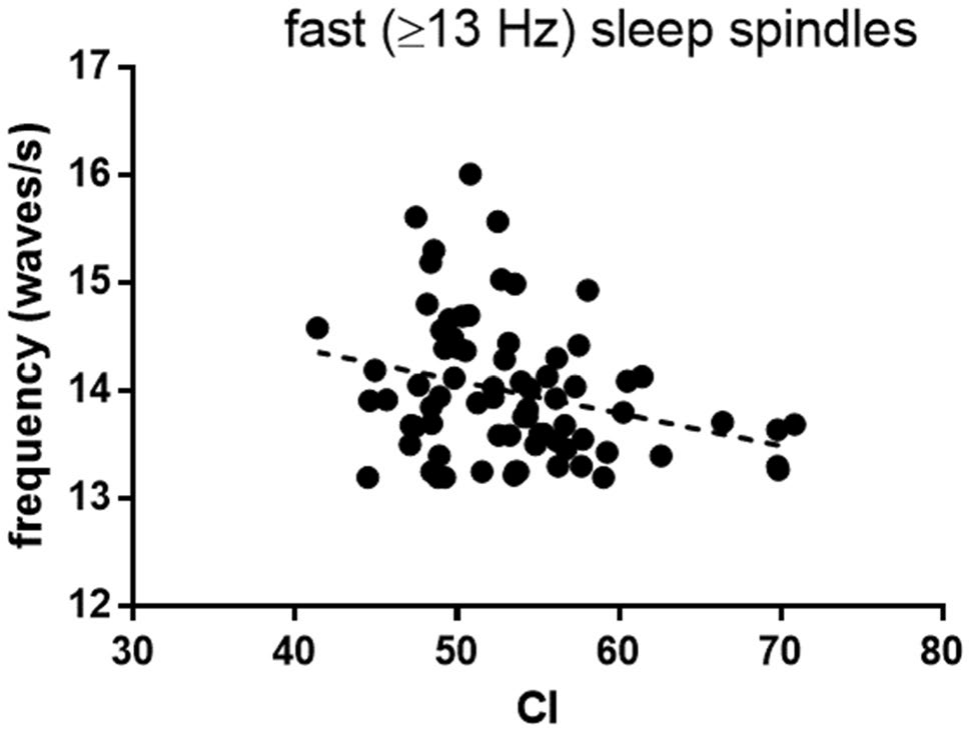
Fast sleep spindle frequency on Fz as a function of CI

On Fz, fast sleep spindle frequency was found to correlate negatively with CI (*r* = − 0.287, *P* = 0.013, Fig. 6). No other sleep spindle variables were found to correlate with CI for fast spindles (*P* > 0.1), slow spindles (*P* > 0.3), nor across all spindles (*P* > 0.2).

On Cz, no sleep spindle variables were found to correlate with CI for fast spindles (*P* > 0.2), slow spindles (*P* > 0.6), nor across all spindles (*P* > 0.5).

## Discussion

A high cephalic index (CI) indicates a short skull and flat face (brachycephaly) in the dog. We found that CI is associated with several sleep-physiological variables. Primarily, effects were observed on the REM sleep phase, with both macrostructure and spectral profile being affected. Shorter-headed dogs spent more time sleeping, and within sleep, more time was spent in REM than non-REM, which is surprising, because usually the opposite is true, with the first two stages of non-REM dominating in, e.g., adult human sleep (Carscadon and Dement 2000). Macrostructure findings were confirmed for both absolute and relative measures of duration; the latter was a control for minor variations in the duration of the recordings. The REM sleep phase of brachycephalic dogs exhibited less relative beta and more relative delta power compared to dogs of lower CI. The effect on beta did not seem to be explained by the overall longer lasting REM phase but was only detectable over the frontal electrode. In non-REM sleep, only the intrinsic frequency of fast frontal spindles was found lower with increasing CI.

Research on the sleep of brachycephalic dogs has so far mainly focused on the propensity of these breeds for sleep apnea (Pratschke 2014). In humans, this condition is associated with increased daytime sleepiness (Gabryelska and Białasiewicz 2020), which may explain the here observed longer sleeping times for brachycephalic dogs. Moreover, the present findings are the first results to show that the sleep of more brachycephalic breeds is also characterized by functionally relevant brain activity differences.

The literature offers two, not mutually exclusive, explanations for why we should expect sleep physiology to be altered by breeding for brachycephaly. First, and most straightforward, anatomical studies have revealed that brachycephalic dogs display anatomical distortions in the brain on different levels of organization (Schmidt et al. 2015; Czeibert et al. 2020; Rusbridge and Knowler 2021), which we can expect to also be expressed in sleep-dependent brain activity as the result of more general differences in brain function and health, but also due to effects on breathing (Barker et al. 2021; Gleason et al. 2022; Mitze et al. 2022; Niinikoski et al. 2023) and (respiratory) thermoregulation (Davis et al. 2017; Gallman et al. 2023) that affect sleep quality. The role of these conditions finds no direct support here, however, since the number of awakenings did not correlate with CI. The propensity for sleep apnea associated with canine brachycephaly (Pratschke 2014) may contribute to some anatomical changes or add to their effect on the brain. This possibility is discussed with regard to how sleep apnea may affect humans [see, e.g., Ahuja et al. (2018)] and is apparent from the memory impairments reported for this condition (Wallace and Bucks 2013; Lee et al. 2016). Second, at least some brachycephalic breeds likely acquired their traits due to breeding for more paedomorphic features, which elicit a caring response in humans (Hecht and Horowitz 2015). This could work via the same selection mechanisms which played a role during initial domestication and are associated with more juvenile features across appearance, physiology, and behavior (Leach et al. 2003; Pörtl and Jung 2019). Under this second hypothesis, we specifically expect patterns associated with juvenile (sleep) physiology.

The combined observation of higher delta power and lower sleep spindle intrinsic frequency in more brachycephalic breeds matches with the literature on sleep EEG correlates of white-matter loss in humans (Sanchez et al. 2019, 2020). Specifically, this pattern could reflect the white-matter loss for which brachycephalic dogs are reported to be at higher risk (Schmidt et al. 2015). Still, some important differences need to be taken into account between our results and the human findings before an analogy is embraced prematurely. First, in humans, both delta power and spindle frequency are correlated with white-matter loss only within a patient population. We instead find the effect to emerge across breeds of presumably healthy dogs. Since in dogs, genetic variation is stronger between breeds than within breeds (Bannasch et al. 2020), this may affect the visibility of the effect compared to human samples. Second, we cannot exclude that the increased REM delta in our sample is linked to increased total REM duration, while in humans, it is also non-REM delta which was compared with white-matter loss (Sanchez et al. 2019) and showed no effect here. Why delta activity increases with more pronounced white-matter damage is not conclusively established and contradicts expectations based on results from young and aging humans (Carrier et al. 2011; Piantoni et al. 2013). Among the explanations offered by Sanchez et al. (2019) is the proposition that delta synchrony is a cortical default state (Sanchez-Vives et al. 2017) enhanced when the cortex suffers de-afferentiation as a result of injury. The decrease in spindling frequency is more comparable with the human findings. Both are significant for frontally recorded sleep spindles. However, Sanchez et al. (2020) do not differentiate sleep spindles into slow and fast, while we report an effect specific to the fast sub-type.

EEG-related observations with potentially functional significance derive from the spectral profile of the REM sleep phase. High beta and low delta power during REM are associated with higher intelligence in human females and better learning performance in dogs (Ujma et al. 2017; Kis et al. 2017c). In this sample, higher CI was associated with the reverse pattern, thus corroborating the anatomical findings (Schmidt et al. 2015; Czeibert et al. 2020; Rusbridge and Knowler 2021), which suggests that we should expect a weaker cognitive performance in high CI, brachycephalic dogs. Notably, the association between CI and REM beta power seems independent of total REM duration, while average REM episode length was not linked with CI. A possible relationship between REM duration and REM delta power needs to be further examined, as the latter may not be independent observations. Correlations with delta are also just below the significance level. The meaning of decreased spindling frequency in higher CI dogs is more difficult to interpret. Most spindle–cognition associations in humans, rats, and mice (Eschenko et al. 2006; Cox et al. 2012; Latchoumane et al. 2017) and all so far observed in the dog (Iotchev et al. 2017, 2020a) concern spindle density and post-sleep recall. Intrinsic frequency (the waves/second of an average spindle) is more ambivalent. It is reported more seldomly to correlate with learning performance than density. When an association was observed, it was positive for young subjects (Kuula et al. 2019), but negative in older humans (Guadagni et al. 2020) and older dogs (Iotchev et al. 2020b), in which a higher intrinsic frequency is either a compensation for emergent pathology or by itself reflects the shortening of thalamo-cortical connections (Gaudreault et al. 2017). Importantly, white-matter deterioration does not generally affect spindle properties equally in young and old subjects (Gaudreault et al. 2018). The lack of an association with other spindle variables strengthens earlier findings in the dog (Iotchev et al. 2017, 2019, 2020a, b), which were potentially limited by the breed variability of the samples. The present finding suggests that this is not a concern for breeds distinguished by head shape with regard to key variables like spindle density.

Our results concerning (relative) sleep duration and the ratio of REM to non-REM sleep in turn lend some support to the hypothesis of brachycephalic dogs having more juvenile brains. Not only do young animals sleep longer, but in many altricial species (animals that are born relatively immature), the percentage of time spent in REM is highest during the first postnatal days and hypothesized to be a carry-over from fetal life [see Zepelin et al. (2005)]. As the newborns of humans (Kurth et al. 2015), dogs (Reicher et al. 2021), and rats (Jouvet‐Mounier et al. 1969) progress in their development, REM durations decrease in favor of a more pronounced non-REM sleep stage. The current study alone cannot prove beyond doubt, however, that the higher percentage of REM sleep observed in brachycephalic dogs is a juvenile trait. Relative differences in REM between wolves and dogs, albeit preliminary, suggest a higher proportion of REM in the captive, hand-raised wolf (Reicher et al. 2022) and thus, REM duration as a potential marker of juvenile sleeping patterns needs to be taken with caution. Crucially, early development and maturation in dogs (Reicher et al. 2021) and humans (Kurth et al. 2015) is characterized by spectral changes in the non-REM sleep stage. We did not observe CI-dependent differences in non-REM power for the tested frequency bands. Looking at the number and average length of REM episodes did not conclusively link either to the correlation of CI with total REM length, but for the sample as a whole total REM length and number of REM episodes were correlated positively. This suggests that across dogs, a higher density of REM episodes, also observed in, e.g., human depression (Palagini et al. 2013), underlies longer total time spent in REM. The most plausible explanation for prolonged REM in brachycephalic dogs will need to eventually integrate behavioral findings with the here observed EEG differences between breeds.

The EEG data used here come from a database containing single (first) polysomnography measurements for each dog and without any behavioral manipulation prior to sleep. This was done to avoid experimental manipulations that cause an alteration in sleep characteristics (including macrostructure, EEG spectrum, and spindle parameters) and could thus potentially confound the relationship between CI and default brain activity, which we wanted to examine first. This, however, is a trade-off which poses two limitations. First, we do not control for the "firstnight" effect that dogs experience in novel sleeping places (Reicher et al. 2020); thus, the results may be specific to a setting in which the sleeping place is unfamiliar. Second, there is no direct measure of cognitive performance related to these recordings. Our interpretation of how these dynamics may relate to cognition is based on previous findings in the human and dog literature. Specifically, a high beta, low delta REM profile was found to correlate with post-sleep recall in a smaller sample of dogs (Kis et al. 2017c), but it is not clear how this test-specific outcome relates to general intelligence, which was the correlate of this spectral profile in human females (Ujma et al. 2017). CI, test performances, and polysomnographic data need to be more directly related to each other in future efforts.

A more serious concern is that CI can be expected to correlate with electrode distance to brain surface (see Fig. 3), with electrodes being closer to the brain in more brachycephalic dogs. This may affect the absolute amplitude and power of the signal but cannot explain why correlations with beta and delta on Fz are of opposite directions. As a precaution against the effects of different skull thicknesses, only relative measures of power were compared. Likewise, our sleep spindle detection uses a relative threshold for the amplitude criterion (Iotchev et al. 2017). One particularly pressing concern related to skull thickness is the filtering effect of the skull bone on higher frequencies like beta (but also alpha and mu). This is implied by observations related to the breach rhythm response of the EEG signal in patients with surgically altered skull surfaces (Cobb et al. 1979). The breaching response suggests, however, that rhythms like beta should be attenuated by a thicker skull. We instead observed a higher frontal beta power in dogs with lower CI, whose skull bone under Fz is thicker (Fig. 3), and thus, beta power differences linked to CI do not seem to be explained by the bone’s filtering properties. We should also note, however, that head size and skull thickness can vary greatly among breeds within both brachycephalic and dolichocephalic dogs as well. In the current study, we could not account for such variation (as no MR scans were available for the subjects).

Future attempts to compare CI and sleep physiology could incorporate health and cognitive assessments and (f)MRI scans to address another set of limitations inherent to the present study. Specifically, a direct link from anatomy to sleep physiology can be demonstrated more conclusively, if we can rule out the intermediate effects of mood, which was shown to affect dogs’ sleep macrostructure (Kis et al. 2017a). Neuropathology is often comorbid with depression in humans [see, e.g., discussed in Ross and Rush (1981), Moldover et al. (2004)] and it is currently not known which neural activity patterns in the dog are direct consequences of anatomical changes versus those preceded by comorbid alterations in mood and emotional systems. The simultaneous application of EEG and (f)MRI could be used in the future to specifically test the white-matter hypothesis more directly. Here, the argument, which was presented above, is more indirect, integrating the present findings with the literature.

Overall, the present findings support the notion that artificial selection changes neural substrates of cognition in the dog. Previous work pointing at the anatomical (Schmidt et al. 2015; Czeibert et al. 2020; Rusbridge and Knowler 2021) and behavioral (Horschler et al. 2019) indications for this process is now complemented with activity from the living dog brain, measured during periods of rest and sleep. The evidence jointly points to neuro-cognitive limitations for more brachycephalic dogs. During sleep, these reflect in both structural and spectral changes of the REM sleep stage. The EEG profile suggests that correlations with CI most likely reflect the white-matter loss reported for brachycephalic breeds.

## Data Availability

The dataset used and/or analyzed during the current study will be made available by the corresponding author upon reasonable request.
